# Rapid Geometric Evaluation of Transportation Infrastructure Based on a Proposed Low-Cost Portable Mobile Laser Scanning System

**DOI:** 10.3390/s24020425

**Published:** 2024-01-10

**Authors:** Haochen Wang, Dongming Feng

**Affiliations:** 1 Key Laboratory of Concrete and Prestressed Concrete Structures of the Ministry of Education, Southeast University, Nanjing 210096, China; wanghc@seu.edu.cn; 2 School of Civil Engineering, Southeast University, Nanjing 210096, China; 3 National and Local Joint Engineering Research Center for Intelligent Construction and Maintenance, Nanjing 210096, China

**Keywords:** transportation infrastructure, geometric measurement, mobile laser scanning, low-cost, 3D reconstruction, data fusion, visualization

## Abstract

Efficient geometric evaluation of roads and tunnels is crucial to traffic management, especially in post-disaster situations. This paper reports on a study of the geometric feature detection method based on multi-sensor mobile laser scanning (MLS) system data. A portable, low-cost system that can be mounted on vehicles and utilizes integrated laser scanning devices was developed. Coordinate systems and timestamps from numerous devices were merged to create 3D point clouds of objects being measured. Feature points reflecting the geometric information of measuring objects were retrieved based on changes in the point cloud’s shape, which contributed to measuring the road width, vertical clearance, and tunnel cross section. Self-developed software was used to conduct the measuring procedure, and a real-time online visualized platform was designed to reconstruct 3D models of the measured objects, forming a 3D digital map carrying the obtained geometric information. Finally, a case study was carried out. The measurement results of several representative nodes are discussed here, verifying the robustness of the proposed system. In addition, the main sources of interference are also discussed.

## 1. Introduction

Transportation infrastructure, mainly roads, bridges, and tunnels, is crucial to the movement of goods and people and is transportation’s lifeline [1]. For economic and societal reasons [2], it is crucial to keep it secure and operating normally. Particularly, transportation infrastructure will be significantly disrupted following natural catastrophes such as earthquakes, floods, mudslides, or explosions, which might result in vehicle traffic issues [3], impassability for huge vehicles, etc. Disaster relief resource allocation and post-disaster traffic diversion are complicated by the unpredictability of transportation conditions [4]. Therefore, it is necessary to quickly reassess the transportation infrastructure (including the geometric size) in the post-disaster area [5], quickly open up disaster relief lifelines in the severely affected areas [6], identify the problem sections in the areas not obviously affected, and restore the orderly operation of traffic as soon as possible [7]. In conclusion, the steady operation of regional traffic in both peaceful and post-disaster conditions relies heavily on systematically collecting geometric information on traffic infrastructure.

INSAR (interferometric synthetic aperture radar), remote sensing technology, and UAVs (unmanned aerial vehicles), etc., are some of the current detection methods that can be used for fast inspection of a vast region. Qualitative determinations, such as locating the damage and assessing damage level, are better suited to INSAR [8] and remote sensing technology [9]. Unfortunately, the measuring distance induces high measurement error, making it hard for quantitative evaluation of small objects [10]. UAVs can quickly cover a broad area from all angles and obtain high-resolution photographs from close range, realizing the object’s three-dimensional measurement [11]. However, data accuracy depends on the reliability of the positioning system and IMU [12], and the mass image demand of tilt photography modeling greatly increases flight time [13], reducing the efficiency advantage. In addition, because of their limited interior volume and signal attenuation [14], tunnels present unique challenges for all of the aforementioned measurement techniques.

Mobile measurement systems [15,16,17] use moving platforms, such as vehicles, as their base of operations and incorporate a wide range of sensors, such as laser scanners, cameras, and GNSS receivers. With a large scan area, quick scan time, and high level of automation [18,19], they can accurately pinpoint an object’s coordinates, as well as determine its geometric outline and intensity [20]. Therefore, vehicle mobile measurement systems are most suited to the data measurement of large-scale banded settings [21] like roads, tunnels, etc. 

Currently commercially available vehicle mobile measuring systems [22,23] include the Street Mapper 360 and VLMS systems. However, all the commercialized systems are of high cost and integrated in the form of inspection vans, which means that all functions (such as road technical condition detection) must be conducted jointly [24]. Due to the specific nature of this paper’s geometric analysis, it would have been wasteful to invest in an inspection vehicle. A low-cost mobile measurement system was more suitable, which also needed to be portable enough to allow travel and be mounted on different kinds of vehicles, further improving its economy.

For transportation infrastructure geometric feature detection based on MLS, Puente et al. [25] extracted the road boundary line and measured the height between the road surface and the top of the tunnel based on vehicle-mounted LiDAR data; the algorithm’s extracted clearance error was less than 1% at the end. To build a closed graph, Ibrahim [26] first distinguished between ground and non-ground points based on density differences, then looked for refined ground points via the neighborhood, and finally detected edge points. Candidate border spots were identified using elevation features, slope variations, and density characteristics of the boundary in the published literature [27,28,29]. Gargoum et al. [30] used vehicle trajectory data to filter ground points and calculated the vertical clearance based on the clustered different types of objects after voxelization of point cloud data. The aforementioned studies provide reliable criteria for the geometric measurement of highways and tunnels. In this work, an economical solution and the further utilization of measurement results were taken into consideration. 

This paper proposes a procedure to extract the geometrical morphology of roadways and tunnels, based on the design of a low-cost and portable mobile measuring system using laser scanning as well as its accompanying software, and took into account the automatic visualization of results to create a digital map of the area under study. Section 2 provides an overview of the measurement system design; Section 3 details the geometric assessment process; Section 4 illustrates how the results can be visualized; and Section 5 introduces a case study and discusses the proposed system’s reliability and the influencing factors. The paper concludes in Section 6.

## 2. Design of the Proposed Measuring System

### 2.1. Instruments Selection and Composition of Measuring System

The measuring system consisted of LiDAR, GPS, and IMU equipment and was designed to work on a moving vehicle. With its advantages in distance and 2D contour measurement, single-line LiDAR was a technically and economically viable option for the objective of this study, which was to measure a roadway and tunnel cross section. There was an acceptable measuring range of road width between 3.75 and 20 m, as the width of a single lane is 3.75 m, and highways with more than three lanes in one direction typically have separators. Therefore, a LiDAR with a measuring range of 25 m was selected. The location data of the system had to be applied to the 2D data from the single line LiDAR to generate 3D point clouds. Hybrid navigation equipment was chosen; it acquired GNSS and IMU data simultaneously with a positional accuracy of 0.02 m, a velocity accuracy of 0.02 m/s, and an attitude accuracy of 0.09°. Two antennas were equipped to receive positioning data. The equipment types are displayed in Table 1.

**Figure 1:**
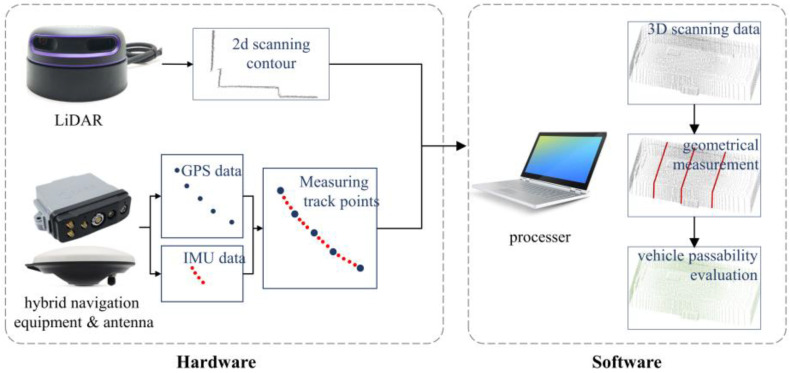
Design and installation of the system.

The working principle of the proposed measurement system is depicted in Figure 1. The single-line LiDAR can capture point cloud data of a 2D plane that reflects the contour of scanning objects, while the position of each 2D contour cannot be confirmed by LiDAR alone. Two approaches are used to record the position of the vehicle. IMU records the attitude and velocity of the vehicle at a high frequency, but a huge cumulative error occurs at long distances, while GPS gives exact data but has a relatively low recording frequency, so it may lack the necessary data to match the moving track [31]. Hybrid navigational tools were chosen to realize the benefits of both methods, providing both position coordinates and attitude data of the system. The IMU module in hybrid navigation equipment supplied velocity and attitude data to compensate for the fitting of track points when there was a lag between GPS records. Moreover, in case of GPS signal loss, the IMU data were also used to make up for the missing position coordinates. The 2D point cloud and positioning data were separately supplied to the processor to produce 3D scanning data and retrieve the geometrical parameters. The data acquisition accuracy of the proposed system is determined by the product parameters of the LiDAR and the hybrid navigation equipment, as described in the previous paragraph.

### 2.2. Design and Installation of the System

**Figure 2:**
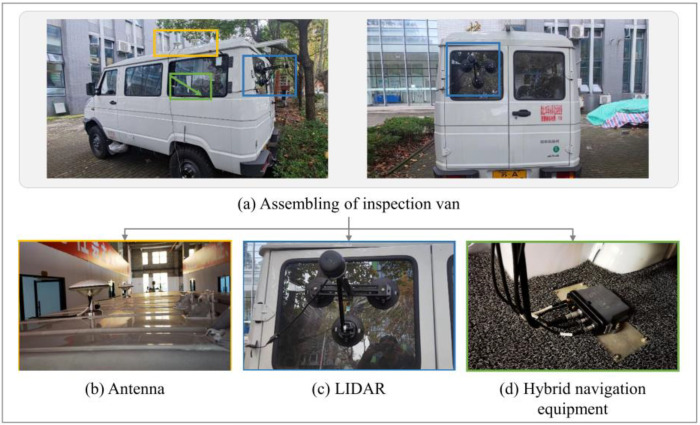
Assembling of the proposed measuring system.

The measurement tool needed to be low-cost, portable, and simple to assemble to meet the need for emergency inspection and increase its usability. The equipment specified in Section 2.1 came with specialized adaptation pieces ready to build and fix, aiming to fulfill measuring precision and economic application. The assembling is depicted in Figure 2. To avoid blocking and maximize the point density, the LiDAR was vertically installed on the back of the vehicle; this configuration also enabled scanning of all lanes in a direction orthogonal to the vehicle’s movement, which streamlined data processing. The LiDAR was mounted on the back glass using a suction cup-equipped triangle bracket; the sensor was bolted to the bracket; in this way, the relative position between the LiDAR and the vehicle (or the navigation equipment) was unchanged during the measurement. The suction cups guaranteed a secure connection and a quick setup, while the triangular bracket’s preset dimensions guaranteed that the LiDAR was mounted upright. The antennae were fastened on the roof by the magnet, joining hybrid navigation equipment placed in the vehicle. All wires and cables were routed through the open window, and the interface was given special treatment to withstand the elements (rain, snow, sandstorms, etc.).

The test results showed that the gadget can be erected and ready to use in 15 min, with a total weight of less than 5 kilos, and disassembled in just 5 min.

## 3. Cloud Point Feature-Based Geometric Evaluation

### 3.1. Overall Procedure

**Figure 3:**
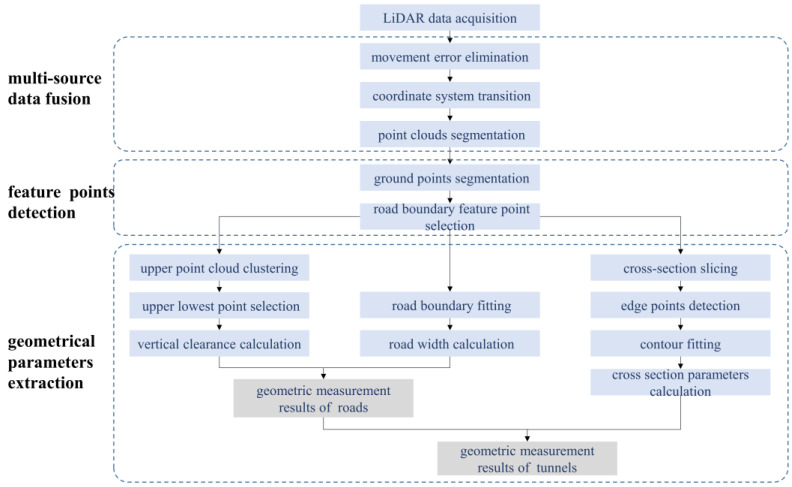
The overall procedure of geometric evaluation.

The entire data analysis and geometric evaluation process is depicted in Figure 3. Multi-source data fusion was first performed, including eliminating LiDAR movement error, reconstructing, and simplifying 3D point clouds from the data acquired by LiDAR, IMU, and GNSS. Suitable measurement frequencies were selected to ensure that data from multiple devices could be matched by the timestamps. Next, a geodetic coordinate system was used to unify the coordinate systems of various devices and build a 3D point cloud of the scanned item. The varying road curvature and undulation complicated features like road cross-section extraction. To improve the efficiency and accuracy of data processing, space coordinate transformation was carried out to convert curved point clouds to straight line point clouds. The horizontal alignment was reflected by tracing points of the vehicle.

To streamline post-scanning processing, the data were manually categorized into road and tunnel types simultaneously when scanning. Based on their positions, the filtered point cloud was then further classified. Based on their positions, the filtered point cloud was then further classified. While road data were separated into upper and ground point clouds, tunnels additionally needed section point cloud extraction.

Taking fixed-length point cloud data as a processing unit, the ground point cloud’s region of interest (ROI) was extracted to remove unnecessary information. Geometric features were used to extract feature points that stood in for the edge of the pavement. Linear equations of road boundaries were used to determine the road’s width. Overhead objects were grouped into clusters to eliminate individual points, and the road clearance limit was defined as the vertical distance between the lowest point of an overhead object and its equivalent ground point. Cross-section size is a more significant factor for tunnels. The outline of the cross section was fitted based on the extracted corner points, which were determined using the curvature change.

### 3.2. Fusion of Multi-Source Data from Integrated Scanning System

#### 3.2.1. Elimination of System Movement Error

During the measuring procedure, the scanning equipment followed the vehicle and maintained a constant distance while an interval of time passed between the sending and receiving of laser points. Therefore, each laser point was generated at a place slightly different from the actual one; when the scanning frequency was low or the movement speed was fast, mistakes induced by the movement could not be disregarded. In addition, the jounce of the vehicle enhanced the errors. An elimination process was conducted to calibrate the original LiDAR data based on vehicle attitude change data collected by the hybrid navigation equipment.

**Figure 4:**
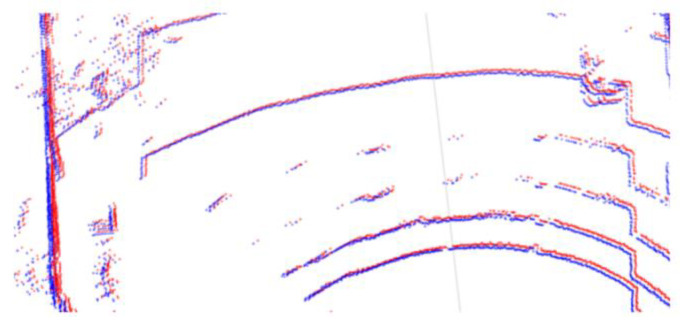
Result of movement error elimination of point clouds.

The vehicle attitude change comprised changes of position, pitch angle, and roll angle. Since LiDAR operates at a fixed frequency, the rotation period was short enough that the system’s attitude changed linearly in every period. This allowed the specified hybrid navigation equipment, whose collecting frequency was up to 100 Hz, to capture velocity and attitude data. First, we obtain the IMU data for the beginning and finish of the current frame’s (one rotation’s) worth of LiDAR data. Then, we define the scanning period as ∆*t*; the change of system position as ${\left(∆x,∆y,∆z\right)}^{T}$, the change of pitch angle and roll angle as ∆*α* and ∆*β*. For scanning point *p_i_*(*x_i_*,*y_i_*,*z_i_*) at a LiDAR rotating angle of *θ_i_* in this period, the attitude change can be calculated by the following equation:

(1) $$∆T_{i}=\frac{2\pi -\theta_{i}}{2\pi }{\left(∆x,∆y,∆z\right)}^{T}$$

(2) $$∆\alpha_{i}=\frac{2\pi -\theta_{i}}{2\pi }∆\alpha$$

(3) $$∆\beta_{i}=\frac{2\pi -\theta_{i}}{2\pi }∆\beta$$

in which $∆T_{i}$, $∆\alpha_{i}$, and $∆\beta_{i}$ separately represent the change of position, pitch angle, and roll angle at scanning point *p_i_*. The revised data *p_i_*′(*x_i_*′,*y_i_*′,*z_i_*′) can be calculated as Equations (4) and (5). Figure 4 shows the result of movement error elimination, in which the red points are the original data, and the blue points represent the corrected data.

(4) $${\left[x_{i}^{\prime },y_{i}^{\prime },z_{i}^{\prime }\right]}^{T}=R_{i}{\left[x_{i},y_{i},z_{i}\right]}^{T}+∆T_{i}$$

(5) $$R_{i}=\left(\begin{array}{ccc} cos∆\alpha_{i} & 0 & sin∆\alpha_{i}\\ -sin∆\alpha_{i}sin∆\beta_{i} & cos∆\beta_{i} & cos∆\alpha_{i}sin∆\beta_{i}\\ -sin∆\alpha_{i}cos∆\beta_{i} & -sin∆\beta_{i} & cos∆\alpha_{i}cos∆\beta_{i} \end{array}\right)$$

#### 3.2.2. Coordinate System Unification of Data

The integrated measurement system keeps track of its data in several coordinate systems. Data from a LiDAR sensor are in a spherical coordinate system, data from an IMU sensor are in a rectangular coordinate system with the system as the coordinate origin, and data from a GNSS sensor are in a WGS-84 coordinate system. To fuse data from multiple sources into a single 3D point cloud, it is necessary to transform the data from each source into a common coordinate system.

However, the disordered, chaotic, and huge volume of data made point cloud analysis more challenging, as it required many adjacent point search operations for registration, filtering, grouping, and segmentation. In addition, challenges in extracting geometrical properties were posed by the varying road curvature and undulation. The point cloud was recreated to reduce its shape, which increased processing efficiency and extraction accuracy.

The *X*′, *Y*′, and *Z*′ coordinate system is defined at the local level. The *X*′ axis represents the direction the vehicle is traveling, the *Y*′ axis is horizontally orthogonal to the *X*′ axis, and the origin represents the position from which tracking points are initiated. The reconstructed point clouds in the local coordinate system $X^{\prime }Y^{\prime }Z^{\prime }$ is always linear along the *X*′ axis, significantly reducing geometric complexity.

**Figure 5:**
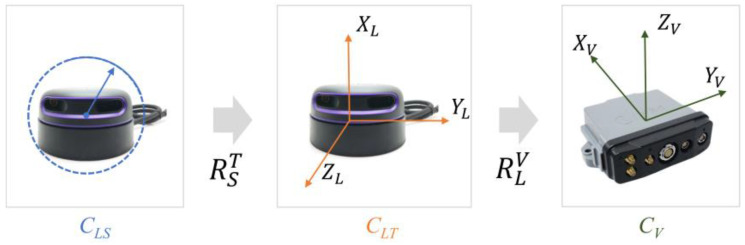
The transition process of multi-coordinate systems.

Figure 5 depicts the conversion process. The original polar coordinate system of the LiDAR *C_LS_* is first transformed into a rectangular coordinate system *C_LT_*, with the origin preserved. $R_{S}^{T}$ is the transition matrix. Second, the LiDAR coordinate system *C_LT_* is converted into the vehicle’s local coordinate system *C_V_*. The converted coordinates ${{(x}_{V},\;y_{V},z_{V})}^{T}$ is calculated by Equation (6). ${{(x}_{L},\;y_{L},z_{L})}^{T}$ is the converted rectangular coordinates of *C_LT_*, in which $x_{L}$ is always equal to 0 since the LiDAR only acquires 2D information in the $Y_{L}-Z_{L}$ plane. $R_{L}^{V}$ is the rotation matrix of converting *C_L_* to *C_V_*, and ${\left(x_{L}^{V},\;y_{L}^{V},z_{L}^{V}\right)}^{T}$ is the coordinate of the origin point of *C_L_* in *C_V_*, representing the spatial relationship between the LiDAR center and the geometric center of the integrated navigation equipment. At the starting point of a road section in measurement, the axis direction of *C_V_* is the same as the local coordinate system $X^{\prime }Y^{\prime }Z^{\prime }$, the reconstructed coordinates $({x^{\prime },y^{\prime },z^{\prime })}^{T}$ may be acquired by Equation (7), in which ∆x is the straight-line distance between two tracking points.

**Figure 6:**
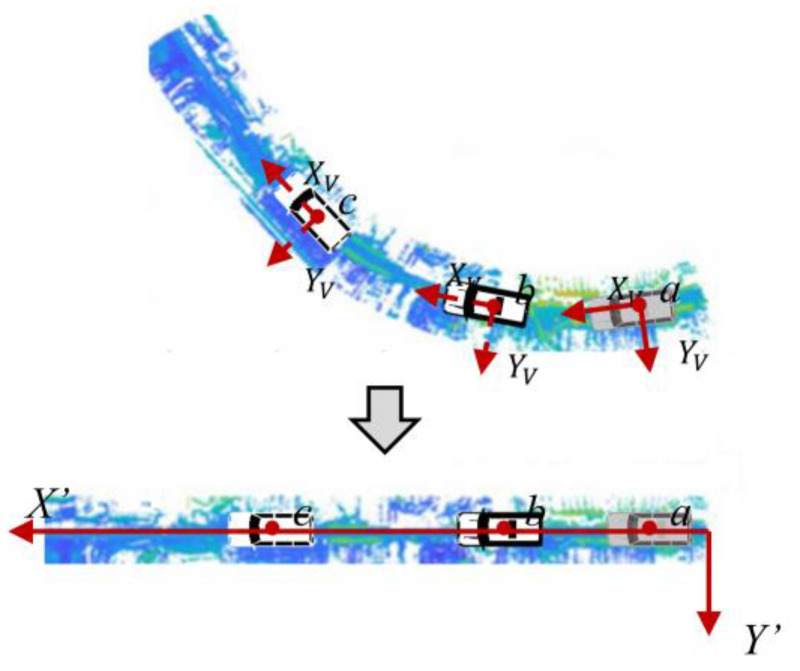
Result of point clouds reconstruction.

The final converted image is shown in Figure 6. It is clear that only the geometry on the $Y_{L}-Z_{L}$ plane stays unmodified after reconstruction. This is reasonable because point cloud data were mostly used in this article to extract cross-section geometric information. The horizontal alignment, another crucial geometrical element, can be reflected by positioning data.

(6) $${{(x}_{V},\;y_{V},z_{V})}^{T}=R_{L}^{V}{{(x}_{L},\;y_{L},z_{L})}^{T}+{\left(x_{L}^{V},\;y_{L}^{V},z_{L}^{V}\right)}^{T}$$

(7) $$({x^{\prime },y^{\prime },z^{\prime })}^{T}={{(x}_{V},\;y_{V},z_{V})}^{T}+{\left(∆x,\;0,\;0\right)}^{T}$$

### 3.3. Feature Points Detection of Road Boundary

**Figure 7:**
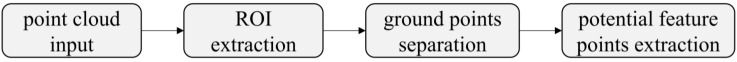
Feature points extraction process.

Due to LiDAR’s wide scanning area, the resulting point clouds contained a great deal of extraneous information; removing this data sped up the processing of the collected information. The steps required to obtain feature points are depicted in Figure 7. Based on a fixed-length road section as a calculating element, feature points were selected using geometric features to differentiate pavement and other objects, which was the basis of geometric measurement.

#### 3.3.1. Rough Extraction of Reign of Interest (ROI)

**Figure 8:**
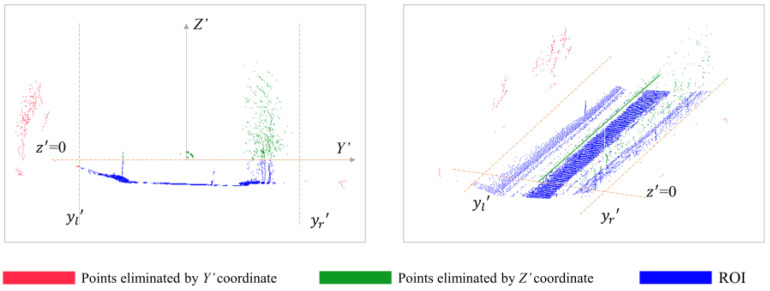
Extraction of ROI.

Straight-through filtering in the width and altitude direction was conducted to preliminarily eliminate sparse point clouds in the distance and expedite the effectiveness of future algorithms. The filter parameter in the width direction was decided with a reign $\;[{y_{r}}^{\prime },\;{y_{l}}^{\prime }]$ referring to the actual distribution of point clouds. Points higher than the LiDAR, such as points with *Z*′ coordinates above 0, were also eliminated as redundant data in the altitude direction. Figure 8 shows that most farther away point clouds were removed after this step; only points adjacent to the road were reserved, defined as ROI.

#### 3.3.2. Further Separation of Ground Points

The filtered point clouds still consisted of dense parts but were morphologically distinguished from the pavement, such as nearby vegetation and the sidewalk. Point cloud plane morphology helped differentiate between the ground and irrelevant objects on the ground, such as cars and trees, because the curvature of road point clouds often varied flatly and consistently. The road was rarely in a single plane over a wide area; therefore, a segmentation method across a narrow range was favored. Assuming that the ground slope in each segment does not vary greatly and can be approximated as a plane, the point cloud is separated into multiple segments with a fixed gap in the *X*′-axis direction. The point cloud is then separated into ground point sets *P_on_* and non-ground point sets *P_off_* using a plane fitting method based on a random sample consensus (RANSAC) algorithm for each fragment.

**Figure 9:**
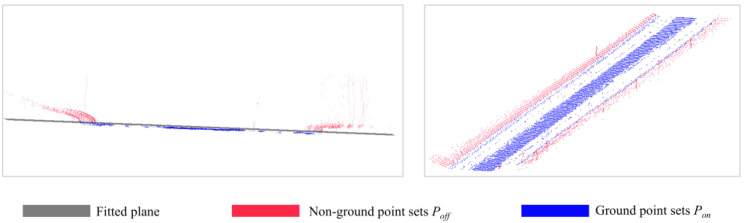
Separation of ground points.

When removing non-ground points and reducing the amount of data for boundary fitting is the purpose, high accuracy is not necessary for the initial stage of ground point separation, and the resulting ground points are rather broad, containing possible points required for the subsequent stage. As shown in Figure 9, even when the separation procedure was complete, more processing was required to extract feature points identifying road boundaries.

#### 3.3.3. Geometric-Based Road Boundary Extraction

Extraction of roadside feature points requires knowledge of the road’s geometry [32]. Considering the influence of system measurement errors and environmental interference, a combination of several geometric features and wide threshold values was proposed to improve the recognition accuracy of road edges as much as possible.

**Figure 10:**
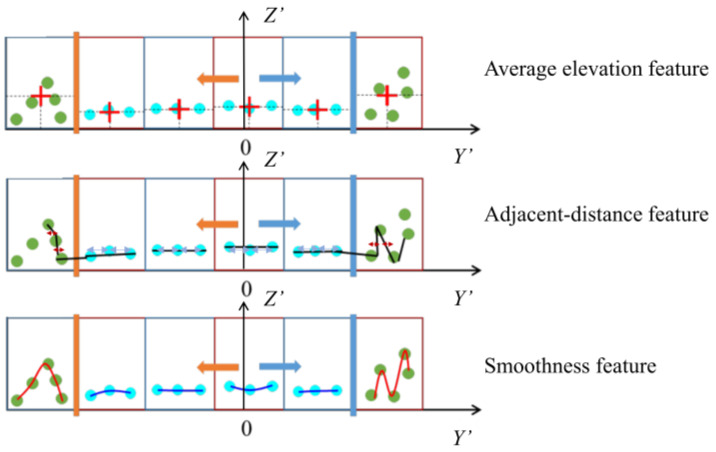
Geometric features used for road boundary extraction.

Selecting geometric features significantly differentiating the pavement and the non-pavement was essential for accurate road boundary identification, and the threshold had to be suitable to filter out impediments without causing false positives. In contrast to non-pavement sites, pavement points exhibited modest elevation change, minimal fluctuation degree, and comparable distance between neighboring points. As Figure 10 illustrates, the sliding window method separates every set of points at a fixed *x*′ coordinate into several grids and analyzes the following three geometric properties along the *Y*′ axis.

##### Average Elevation Feature

The average elevation of points in each grid was calculated. Since the average elevation in the grid containing the road edge was higher than the average elevation in the road surface, an appropriate threshold was determined in accordance with the numerical features to determine the grid where the road edge area was located. Equations (8) and (9) show the filtering rules.

(8) $$T_{height1}\leq Z_{max}-Z_{min}\leq T_{height2}$$

(9) $$\sqrt{\frac{\sum {\left(Z_{i}-\mu \right)}^{2}}{n}}\geq T_{height3}$$

$Z_{max}$ and $Z_{min}$ are the maximum and minimum height values of the points in the grid, respectively; $Z_{i}$ is the height of a single point, μ is the average height value of all points, and *n* is the total number of points. The standard thresholds are $T_{height1}$, $T_{height2}$ and $T_{height3}$ and are based on the curb height of the road. $T_{height2}$ can range in [0.15, 0.35], while $T_{height1}$ and $T_{height3}$ are often in the range of [0.01, 0.05].

##### Smoothness Feature

Since the pavement plane had an obvious difference in smoothness compared with the edge with abrupt elevation, the feature points could be identified by finding the locations with large changes in the smoothness coefficient. The smoothness feature is useful for identifying probable road edges since it accurately reflects the uniformity of laser point distribution in a localized region. The calculation is shown in Equations (10) and (11).

(10) $$s\geq T_{smoothness}$$

(11) $$s=\frac{1}{\left|n\right|\left\|Z_{i}\right\|}\cdot \sqrt{\sum_{\begin{array}{c} Z_{i,j\in n}\\ i\neq j \end{array}}{\left(Z_{i}-Z_{j}\right)}^{2}}$$

$Z_{i}$ is the height of a single point in the grid, s is the smoothness parameter, and *n* is the total number of points. The value threshold $T_{smoothness}$ depends on the road boundary type, with a normal range of [0.001, 0.005].

##### Adjacent-Distance Feature

The laser points along the pavement were evenly spaced along the *Y*′ axis, whereas they showed more noticeable variation around the road’s edge. Therefore, as Equations (12)–(14) show, the horizontal distance threshold between two neighboring points on the same scan line determines the feature points that can be identified at different places.

(12) $$\delta_{gk}=\frac{\sum \left|{Y^{\prime }}_{i}-{Y^{\prime }}_{i+1}\right|}{n-1},\;\;i\epsilon \left[1,n-1\right],\;\;k\epsilon [1,N-1]$$

(13) $${Y^{\prime }}_{gk}=\frac{\sum {Y^{\prime }}_{i}}{N}$$

(14) $$\left\{\begin{array}{c} \delta_{gk}<\delta_{gk+1},\;\;{Y^{\prime }}_{gk+1}\leq 0\;\;\\ \delta_{gk}>\delta_{gk+1},\;\;{Y^{\prime }}_{gk+1}>0 \end{array}\right.$$

$\delta_{gk}$ is the average horizontal distance between neighboring points in one grid, ${Y^{\prime }}_{i}$ is the *y*′ coordinate of any point in this grid, and n is the the number of points in the grid. The average *y*′ coordinate value of a location along a canning line is denoted by ${Y^{\prime }}_{gk}$, where *k* is the grid number, and *N* is the total number of grids in one canning line. Due to the vertical arrangement of LiDAR, the average horizontal distance between adjacent points in a single grid grows with increasing scanning distance, but it does not apply in road edge areas. If the $\delta_{g}$ value of an external grid is less than that of its interior adjacent one, the external grid is considered to be the possible road edge area.

**Figure 11:**
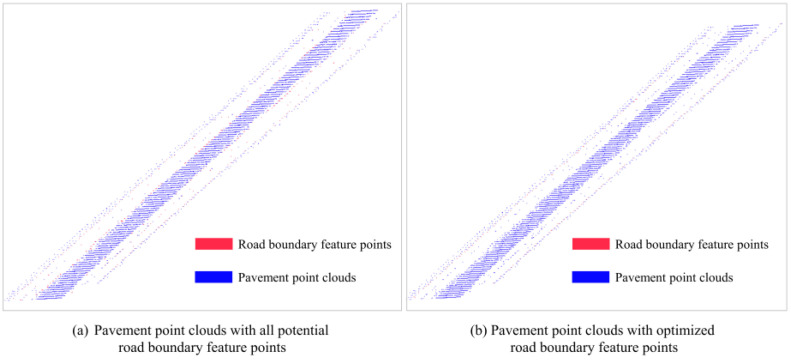
Extraction results of road boundary feature points.

The above characteristics are used to narrow down the list of grids that might contain a road’s edge. Candidate roadside feature sites are selected as those closest to the 0 *y*′ coordinate in the grids that concurrently meet all three feature thresholds. If many grids are available that are satisfactory for a single scan line, the one that is furthest away is chosen. The grid’s width is fixed at 0.1 m to account for the size of the road’s curb stones and the side ditch. The identification result is shown in Figure 11. Figure 11a shows the possible feature points of all qualified grids, and Figure 11b filters them by picking the points in the outermost grids.

### 3.4. Extraction of Geometrical Parameters

#### 3.4.1. Extraction of Road Width

**Figure 12:**
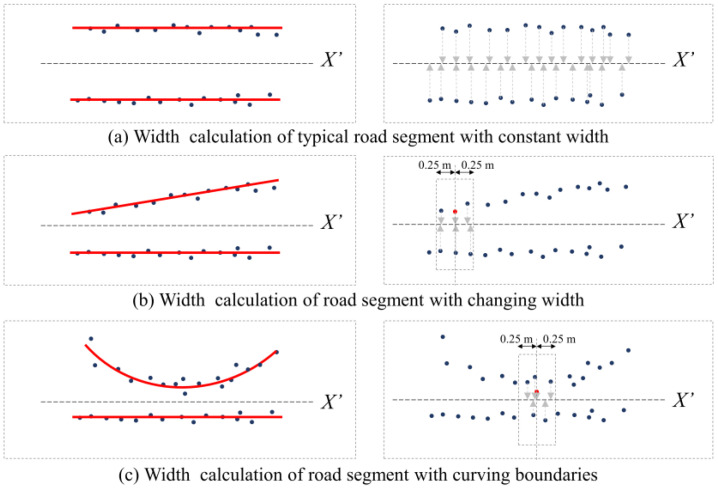
Calculation of road width.

The RANSAC method was used to fit the road boundary through the candidate feature points extracted in Section 3.3.3, and the road width was calculated by measuring the distance between two boundaries. Figure 12 depicts a variety of realizable boundary outputs under varying conditions. Since the *X*′ axis represents the vehicle’s movement down the road, at least one side of the road’s fitted boundaries can be converted to be perpendicular to the *X*′ axis.

#### 3.4.2. Extraction of Vertical Travel Clearance

##### Segmentation of Upper Point Clouds

After the road area was determined using the boundary fitting technique described in the prior chapters, we defined the boundary of the left and right sides of the road as $f_{1}\left(y^{\prime }\right)$ and $f_{2}\left(y^{\prime }\right)$, respectively. The point above the road can be derived through Equation (15). Point clouds below the height of LiDAR can also be filtered using pass-through filtering to increase the calculation’s performance.

(15) $$f_{1}\left(y^{\prime }\right)\leq y^{\prime }\leq f_{2}\left(y^{\prime }\right)$$

##### Vertical Headroom Calculation of Road

Above the ground points, a regional point cloud is clustered using hyper-voxel clustering. After the point cloud is separated into multiple objects, vertical headroom computation is carried out for each object in the cluster. The position ${\left(x^{\prime },y^{\prime },z^{\prime }\right)}^{T}$ of the object’s lowest point is chosen and projected onto the ground plane. The height value $z_{nd}^{\prime }$ on the road surface is extracted by the corresponding plane coordinates ${\left(x^{\prime },y^{\prime }\right)}^{T}$. Then, the vertical clearance constraint $h_{c}$ is determined by Equation (16).

(16) $$h_{c}=z^{\prime }-z_{nd}^{\prime }$$

##### Vertical Headroom Calculation of Tunnel

Since a tunnel is an enclosed structure, the height of its signs and lighting equipment must be considered when determining its vertical headroom. The vertical headroom at the tunnel axis is more relevant than the vertical headroom extracted using the procedures described in the preceding sections, which is the height of the lining at the edge of the pavement. As a result, adjustments need to be made to the region chosen during the segmentation of the upper point clouds. Points outside of a 4 m width (the width of a single lane) in the cross-section direction are discarded to extract the top point cloud, as shown by Equation (17) (the unit is m) with the tunnel axis as the center. As before, the point cloud is divided, and the lowest point is chosen.

(17) $$\frac{f_{1}\left(y^{\prime }\right){+f}_{2}\left(y^{\prime }\right)}{2}-2\leq y^{\prime }\leq \frac{f_{1}\left(y^{\prime }\right){+f}_{2}\left(y^{\prime }\right)}{2}+2$$

#### 3.4.3. Extraction of Cross-Section Contour of Tunnels

**Figure 13:**
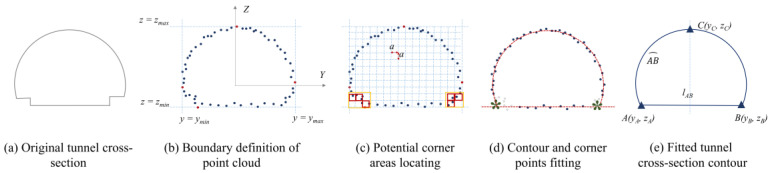
Cross-section measurement of tunnels.

The cross-section cloud points were obtained by sequentially slicing the tunnel in the direction of travel, with the size of the cross section being dictated by the fitted parameter expression of the boundary. Figure 13 depicts the procedure.

1. The laser points of each cross-section slice are distributed on the *Y*′-*Z*′ plane. Pick the maximum and minimum values of *y*′ and *z*′ coordinates as the boundary of the point cloud, as shown in Figure 13b.
2. Grids with a fixed size *a* × *a* are evenly distributed with the boundary, and the normal vectors of all points in each grid are calculated; the areas with large changes in value (determined by a variance threshold) are picked as the potential corner areas in accordance with Figure 13c.
3. The points in the middle of the two possible corners are separated into two contour lines of cross section, and the RANSAC method is used to fit each of those lines into an expression for the parameters. Each corner is defined as the point where two fitted contour lines intersect. As shown in Figure 13d,e, this process yields the necessary parameters for characterizing the cross section’s shape.

In addition, as illustrated in Figure 13a, the shape at the intersection of the roadway curb and the tunnel lining varied widely. Figure 13e shows a simplified version of the detail shape changes, where the pavement is made to directly connect with the lining without any height change, because the paper aimed to extract the overall shape of the cross section. In step 2, the entire curb space along a road is counted as a single candidate corner space to meet the simplicity. As shown in Figure 13c, the possible corner areas are defined as the collection of nearby feature grids due to the high concentration of feature grids with considerable variations of normal vectors in the road curb area.

## 4. Automated Visualization of Evaluation Results

**Figure 14:**
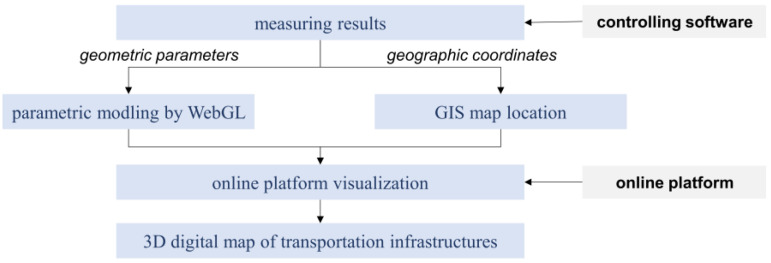
The procedure of automated results visualization.

A self-developed software handled the measuring procedure and output results in near-real time. However, it was difficult to effectively demonstrate and evaluate traffic routes based just on numerical statistics. To create a 3D digital map carrying the gathered geometric information, a web-BIM (building information model) allowed for the depiction of inquiry results linked to an online platform, and the 3D models of measuring items were reconstructed and positioned on the GIS map. A data application programming interface (API) automatically connected the platform’s results to the 3D digital map, as shown in Figure 14.

### 4.1. Parametric Modeling of Measuring Object

#### 4.1.1. Component Library and Description Parameters

This paper’s analysis focuses on the geometric characteristics of the controls rather than the finer points of the measured items, which had no bearing on the overall assessment. A parametric modeling approach [33] was chosen to reconstruct measuring objects with minimal data effectively. By adjusting the description parameters of the unified model to reflect the actual value, this technique created a 3D BIM model of a specific object across all measurement categories [34,35]. Since the geometrical morphology was expected to be constant across a single parametric model, the values of description parameters used in the modeling process had to be kept below the minimum value of the measurement findings.

The component library included unified models for all conceivable measurement items together with description parameters [36], as shown in Table 2. The geometric parameters of roads and tunnels were classed into transverse and longitudinal parameters. The longitudinal parameters described the length and location, whereas the transverse parameters dictated the shape of the cross section. The vertical road clearance was represented by a fixed-height portal frame placed at the start of the road model.

#### 4.1.2. Rapid Modeling Based on WebGL

**Figure 15:**
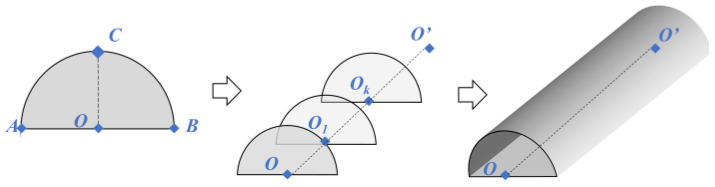
The parametric reconstruction process of tunnels.

A JavaScript package that encapsulates WebGL drawing commands was used to create the visualization. One of the best JavaScript 3D libraries, *Three.js* contains a wide variety of object types in addition to some simple yet effective modeling and interaction tools [37]. Two-dimensional (2D) cross sections can be created using the *ShapeGeometry* tool in Three.js and then extruded along a path specified by the *ExtrudeGeometry* tool according to the longitudinal parameters. Figure 15 describes the process of tunnel reconstruction. Assume that the unit’s origin is at the starting point *O*. The reconstructed model will begin at *O*, the 2D cross section will be established using the parameters in Table 1, and the model will proceed along a straight axis for a distance of length *L* (also from Table 1) from *O* to ending point *O*’.

### 4.2. Automated Visualization of Measurement and Evaluation

#### 4.2.1. Design of Measurement Controlling Software

The measuring system designed in Chapter 2 consisted of equipment from different manufacturers; no existing bundled software could simultaneously process all the data from various sources. A measurement program was written to manage the scanning system’s operations and process the collected data to generate study results. LiDAR data capture, integrated positioning acquisition, and a comprehensive evaluation program were all included in the package. The LiDAR data capture program took in point cloud data and converted it to text files so that it could be accessed by other modules. Textual output of integrated navigation data based on inertial navigation and GPS was the responsibility of the integrated positioning acquisition program. Invoking LiDAR point cloud data and integrated navigation posture data, the evaluation program then extracted the geometric properties of the measured item.

**Figure 16:**
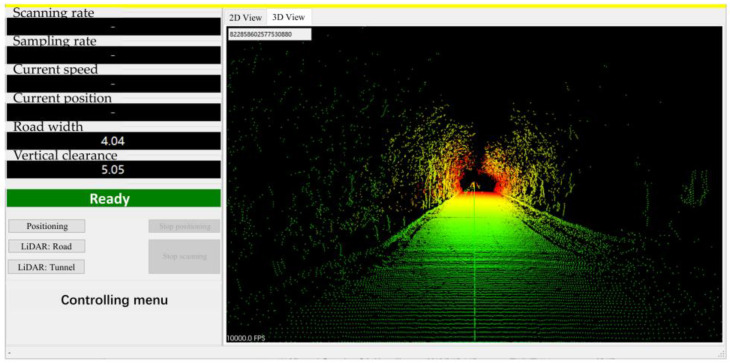
User interface of device controlling software.

The database system used was SQLite, the core framework environment was Net FrameWork 4. The software is compatible with Windows 7 and Windows 10; it requires at least an Core i3 processor (Intel, Santa Clara, CA, USA), 8 GB memory, GeForce 240 graphics card (NVIDIA, Santa Clara, CA, USA), and 2 GB storage space (or other computer configurations on the same level). It processes and maintains the gathered point clouds, enables viewing of the gathered point cloud data, and allows for historical data queries. As shown in Figure 16, point cloud data can be shown in either the LiDAR’s original polar coordinate system or a standard 3D rectangular coordinate system after data fusion. In addition, the program shows details such as the rate of LiDAR scanning and sampling, the current speed and position, the width and height of the road, and whether or not the vehicle is in operation.

#### 4.2.2. Digital Map Development Based Online Platform

Since a large area needed to be covered to evaluate specific traffic routes, the results of separate investigations had to be put into a broader context. An online platform was built using a web-BIM and GIS (geographic information system). The web-BIM enabled the online display of 3D models, which were located on the real map background provided by the GIS [38]. The data gathered were then used to create a digital map showing the precise positions of the measured objects and their geometric properties.

A data API was written to facilitate the rapid generation of a digital map of the object being measured through its seamless integration with the cloud-based service. The resulting digital map of the region can be enhanced or modernized by incorporating the findings of additional surveys. The model can also store data on deformation measurements and disease detections taken at various times, enabling data visualizations for use throughout the facility’s operational lifetime.

## 5. Field Test and Discussion

### 5.1. Test Information

**Figure 17:**
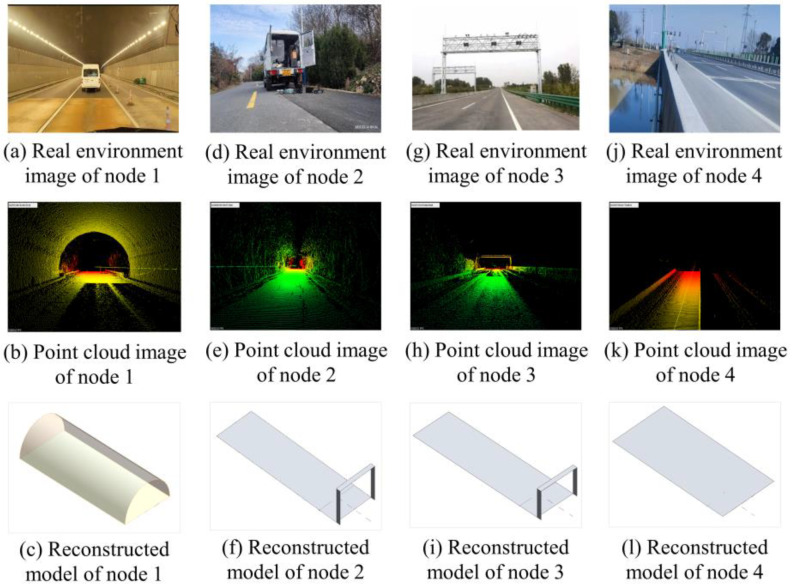
Field test results visualization of typical nodes.

The accuracy of the proposed measuring system was tested in the field along a 10 km stretch of highway. The outcomes of the tests conducted on typical nodes are displayed in Table 3. Figure 17 illustrates the matching real environment photographs, point cloud images, and reconstructed models of nodes selected in Table 3. Figure 17a–c relate to node 1, Figure 17d–f relate to node 2, Figure 17g–i relate to node 3, and Figure 17j–l relate to node 4.

**Figure 18:**
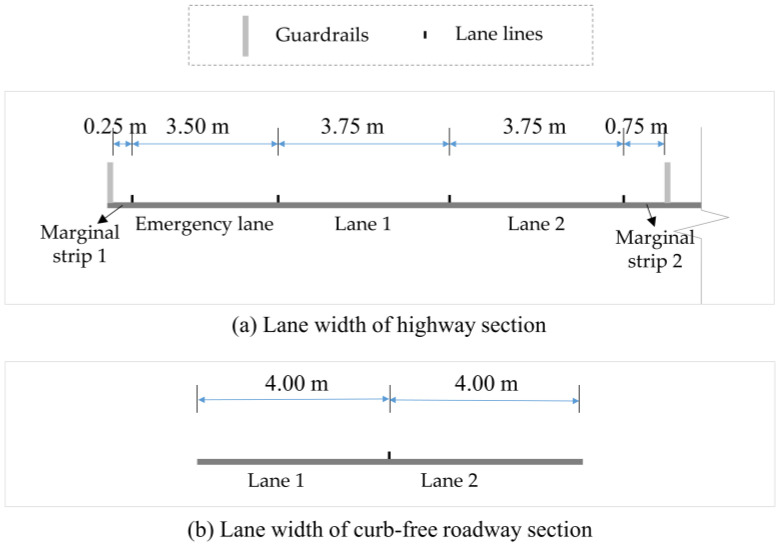
Lane width tape measurement result of typical road sections.

Results were compared to the true value to determine their accuracy. The width of a road can be estimated by counting the number of lanes, since each lane has a standard width of between 3.5 and 3.75 m (11 and 12 feet). A practical tape measurement was conducted at the highway section (node 3–4) and curb-free roadway section (node 2) separately to verify the ground truth value of road width; the results are shown in Figure 18. Especially for the curb-free roadway section illustrated by Figure 18b, because of the uneven edges of pavement, the width of each lane was determined by the average value of multiple measurement cross sections. This work specifies 12 m for a three-lane highway and 8 m for a two-lane city street. The design documents will specify the maximum tunnel height.

### 5.2. Results Analysis

Node 1’s point cloud was gathered under optimum conditions, with full coverage of the tunnel and roadway by laser pointers, and its test findings were very close to the real value. The test results demonstrate the measuring system’s good applicability in various settings, including the one depicted by Node 2’s vegetation- and curb-free roadway, wherein the smoothness feature and adjacent-distance feature outperformed the average elevation feature. The scanning range was obstructed by neighboring vehicles at node 3, a gantry-framed stretch of roadway. Because the pavement point clouds were missing certain information, the road’s edge could be wrongly identified. The result for road width at node 3 was 8.04 m, as the neighboring vehicle was blocking the scan of the third lane of pavement. Because point clouds of the top gantry frame could be obtained reliably at all times, test results for vertical clearance were often constant.

**Figure 19:**
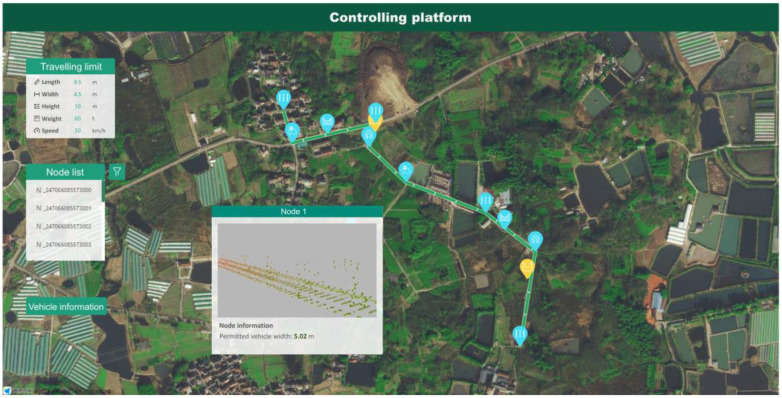
Results visualization of typical nodes.

Node 4’s point clouds, taken from a highway bridge, were also somewhat incomplete, but their test results were closer to the true value than node 3’s. It is easy to see that in node 4’s point clouds, the road guardrails and even the blocked pavement area were reserved more thoroughly than in node 3. As a result, on node 4, the edge of the blocked area was less noticeable than the road guardrail area when it came to boundary features. As the bottom of the road guardrail was obscured at node 3, the geometric features of the road guardrail area and the blocked pavement area were similar due to the lack of laser points. In contrast, the geometric features of the edge between the complete surface and the blocked area were more distinct. The reconstructed tunnel model of node 1 is shown in Figure 17c. Due to the symbolic nature of the model, it is depicted as a straight tunnel with a constant cross-section size. Node 4’s road model is presented as a flat, static plane. A fixed-height portal frame at the beginning of the pavement model represents the travel clearance limits present in the road models of nodes 2–3. The digital map derived from testing data is displayed in Figure 19. The measured objects are represented on the GIS map via nodes. Each measurement node has a drop-down menu to access the point cloud data, reconstructed models, and dimension data.

### 5.3. Discussion of Measurement Accuracy

#### 5.3.1. Influence of Vehicle-Blocking

The test results showed that blocking from nearby vehicles was the primary factor affecting measurement accuracy, especially when vehicles were close together for extended periods. The moving vehicles obscured the point clouds in those lanes, and the point clouds on the side of the vehicle were taken to be the boundary of the road. Therefore, the deviation of the measured road width was often approximate to the width of one lane. Each measurement unit’s road width measurement result was compared to the data of its two neighboring units to reduce the error generated by nearby vehicle obstruction. The measuring units whose results differed from those of their two neighboring units were flagged for further inspection.

#### 5.3.2. Misidentification of Vegetation above the Road Surface

When working with point clouds, vegetation growing above the road surface on tree-lined roadways is often mistaken for overhead obstacles. In contrast to portal frames, tree-lined roadways do not restrict passage in the event of an emergency, regardless of the height of the surrounding vegetation. To further identify the overhead vegetation obstruction from other fixed limitations, the geometry of clustered upper point clouds should be investigated. The shape of fixed overhead restrictions is fairly regular, with surface normal vectors distributed concentratedly. Overhead vegetation point clouds have separate surface normal vectors. The vertical travel clearances brought by overhanging trees are noted separately. In the event of an emergency, the overhead clearance dictated by the fixed constraints is prioritized over the overhead clearance caused by vegetation.

#### 5.3.3. The Environment-Affected LiDAR Data Quality Fluctuation

The quality of LiDAR data affects the authenticity of measurement results directly, including the weather, the light, and the reflection characteristics of the measured object surface. In cases of strong light, heavy rain or snow, the laser reception may be disturbed, inducing a partial absence of point clouds. The variation of the measured object surface also brings a difference in point cloud density. A proper equipment choice helps to weaken this effect; the LiDAR chosen in this article performed well in general conditions for tunnels and pavements of different materials, while it was still restricted in extreme weather.

#### 5.3.4. The Influence of Vehicle Turbulence

The point cloud error caused by vehicle movement can be eliminated through the approach introduced in Section 3.2.1. However, large vehicle turbulence may cause independent vibration of the bracket and LiDAR, whose effects cannot be eliminated by the IMU data of navigation equipment mounted in the vehicle. To minimize turbulence, the vehicle is required to drive within a speed limitation, especially in uneven pavement sections. The speed limitation in ideal pavement sections is set to be 80 km/h.

## 6. Conclusions

1. A lightweight, portable measuring system was designed, which is easy to mount on a vehicle and take down again after use. Compared with existing products, the proposed system serves as a convenient and economical large-area measuring approach, showing good applicability to different vehicles. The unit combined a LiDAR with a positioning system, with the two systems’ data being synchronized through the use of time stamps. To complete the measuring assignment at a lower cost, the equipment was chosen in accordance with the typical geometric scale of transportation networks.
2. A method for extracting geometrical parameters of roads and tunnels using LiDAR data is proposed. The data of various devices are fused to form the 3D pint clouds of measured objects. A combination of three defined geometrical thresholds are used to extract feature points of road boundaries, which reduces the potential randomness of the single-feature method. The geometrical parameters to be measured, including road width, vertical travel clearance, and cross-section contour of tunnels, are noted to be examined accurately.
3. A component library was established, and the measured parameters were classified to define the 3D model of measured objects. The self-developed software accomplished the measuring and data processing, and the measuring results were connected to the online evaluation platform and visualized on a GIS map, forming a 3D digital map of the measured area carrying the traffic capacity information as well as other life cycle operation and maintenance information. Instead of text-formed results, the automated 3D reconstruction enabled the demonstration and analysis of the results in an intuitive and user-friendly way.
4. A 10 km stretch of road was measured in a case study. The outcomes of the tests conducted on typical nodes were discussed to evaluate the accuracy. The results show that the system worked stably in the condition of a full acquisition of point clouds. In ideal situations, the proposed system can bring results at an accuracy similar to existing approaches but more conveniently and economically. However, measurement is also affected by nearby vehicles and the environment, which should be further analyzed to minimize the impact through post-processing approaches.
5. The proposed measuring approach can be applied to related fields, such as architectural mapping, land survey, forest management and planning, and agriculture investigation. The LiDAR-acquired point cloud map helps quickly obtain information about the investigation object, enabling further management in applications such as farmland water supply calculation, vegetation growth prediction, and land reallocation. As the system’s applicability to different fields varies greatly, LiDARs of different parameters and various carriers (vehicle, robot, UAV, etc.) should be taken into consideration.

## Figures and Tables

**Table 1:** Equipment selection of measuring system.

Equipment Name	Equipment Type
LiDAR	RPLIDAR A3, SLAMTEC, Shanghai, China
Hybrid navigation equipment	NPOS220S, BDStar Nvigation, Beijing, China
Antenna	HX-CSX601A, Harxon, Shenzhen, China

**Table 2:** Description parameters of measuring objects.

Title 1		Title 2	Title 3
Road	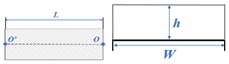	Road width *W*; vertical clearance *h*	Road length *L*, coordinates of starting point *O* and endpoint *O*′
Tunnel	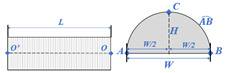	Road width *W*; tunnel height *H*; lining curve formulation *l_AB_*	Tunnel length *L*, coordinates of starting point *O* and endpoint *O*′

**Table 3:** Field test results of typical nodes.

	Node Number	1	2	3	4
Test Parameters (m)					
Road width	Ground truth	12	8	12	12
	Test result	12.05	8.04	8.11	12.06
Vertical clearance	Ground truth	8.05	/	6.5	/
	Test result	7.67	5.05	6.86	/

## Data Availability

Data available on request due to restrictions. The data presented in this study are available on request from the corresponding author. The data are not publicly available due to the policies of the corresponding institute.
